# Volatiles Influencing Sensory Attributes and Bayesian Modeling of the Soluble Solids–Sweetness Relationship in Strawberry

**DOI:** 10.3389/fpls.2021.640704

**Published:** 2021-03-17

**Authors:** Zhen Fan, Anne Plotto, Jinhe Bai, Vance M. Whitaker

**Affiliations:** ^1^Horticultural Sciences Department, IFAS Gulf Coast Research and Education Center, University of Florida, Wimauma, FL, United States; ^2^Horticultural Research Laboratory, USDA-ARS, Fort Pierce, FL, United States

**Keywords:** descriptive analysis, flavor, fruit chemical analysis, sensory, sugars, volatile organic compounds

## Abstract

Descriptive analysis *via* trained sensory panels has great power to facilitate flavor improvement in fresh fruits and vegetables. When paired with an understanding of fruit volatile organic compounds, descriptive analysis can help uncover the chemical drivers of sensory attributes. In the present study, 213 strawberry samples representing 56 cultivars and advanced selections were sampled over seven seasons and subjected to both sensory descriptive and chemical analyses. Principal component analysis and K-cluster analyses of sensory data highlighted three groups of strawberry samples, with one classified as superior with high sweetness and strawberry flavor and low sourness and green flavor. Partial least square models revealed 20 sweetness-enhancing volatile organic compounds and two sweetness-reducing volatiles, many of which overlap with previous consumer sensory studies. Volatiles modulating green, sour, astringent, overripe, woody, and strawberry flavors were also identified. The relationship between soluble solids content (SSC) and sweetness was modeled with Bayesian regression, generating probabilities for sweetness levels from varying levels of soluble solids. A hierarchical Bayesian model with month effects indicated that SSC is most correlated to sweetness toward the end of the fruiting season, making this the best period to make phenotypic selections for soluble solids. Comparing effects from genotypes, harvest months, and their interactions on sensory attributes revealed that sweetness, sourness, and firmness were largely controlled by genetics. These findings help formulate a paradigm for improvement of eating quality in which sensory analyses drive the targeting of chemicals important to consumer-desired attributes, which further drive the development of genetic tools for improvement of flavor.

## Introduction

The garden strawberry (*Fragaria* × *ananassa*) is popular for its pleasant aroma and sweet taste. High levels of sweetness and intense flavor are the leading factors driving frequent strawberry purchases (Colquhoun et al., 2012). This is consistent with consumer sensory studies that identify sweetness intensity and flavor intensity as the top sensory attributes associated with consumer liking (Jouquand et al., 2008; Schwieterman et al., 2014). Therefore, sweetness and flavor must be essential criteria during all stages of strawberry breeding in order to develop cultivars that are successful in the marketplace.

While a consumer panel is useful for revealing relationships between some sensory attributes and hedonics (Oliver et al., 2018a), it requires a large number of panelists due to variation from diverse demographic backgrounds (Knee, 2002). In contrast, a descriptive analysis (DA) only requires eight to 12 trained panelists. A DA begins with creation of descriptors for the product and a process to calibrate the descriptors with reference standards until precise and specific descriptors are achieved (Lawless and Heymann, 2010). While a DA does not directly quantify hedonic responses, it can be used to interpret consumer liking when the same samples are tested by consumer panels (Lawless and Heymann, 2010). Trained DA panels have been widely implemented for sensory evaluations of fruits and vegetables under different storage conditions, maturity stages, spans of postharvest storage, or cultural practices (Cliff et al., 1998; Varela et al., 2005; Kårlund et al., 2015).

To identify the chemical drivers of sensory attributes, DA can be combined with chemical analysis. Combined analyses have led to discoveries of the relationships among sensory intensities and flavor-active compounds. In tomato, sweetness, sourness, bitterness, astringency, and saltiness intensities were found to be correlated to sugars, acids, and volatiles (Baldwin et al., 1998). In peach, sweetness and aroma are influenced by organic acids, sugars, and acids (Colaric et al., 2005). In jujube and many other fruits, multiple volatiles contribute to fruity flavor (Stavang et al., 2015; Plotto et al., 2017).

Flavor is a complex of inputs from multiple senses. The range of chemicals contributing to the flavor of fruits includes those interacting with taste buds like sugar and acids and those interacting with olfactory receptors like volatile organic compounds (Lim and Johnson, 2012; Klee and Tieman, 2018). Among all sensory attributes, sweetness is the predominant driver for consumer preference in strawberry (Schwieterman et al., 2014). However, the majority of strawberry consumer samples fail to meet consumers’ expectations of sweetness according to our recent consumer sensory study (Fan et al., 2020). It is well established that congruent odors increase taste sensations (Frank and Byram, 1988; Lim and Johnson, 2012), and specific volatiles can enhance sweetness perception (Baldwin et al., 2004, 2008; Tieman et al., 2012). Therefore, increasing sweetness-enhancing volatile content in fruit through horticultural practices or genetic manipulations is an attractive alternative to increasing sugar, as it should not have a detrimental effect on agronomic traits like yield (Whitaker et al., 2012). In the same consumer study (Fan et al., 2020), we found 20 volatiles enhancing sweetness perception independently of sugars. Adding volatiles to the predictive model explained 28% more variability in sweetness than a model with sugars and acids alone (Fan et al., 2020). Additional sensory-chemical studies are needed to validate the effects of sweetness-modulating volatiles and evaluate volatiles’ effects on additional sensory attributes (Whitaker et al., 2020).

Sugars, acids, and minerals are the major soluble components in strawberry fruit. Sucrose, glucose, and fructose together account for 99% of total sugars (Montero et al., 1996). Soluble solids content (SSC) has long been considered a good approximation of total sugars in strawberry fruit (Pelayo-Zaldívar et al., 2005; Whitaker et al., 2011). The high-throughput capability of refractometers allows SSC to be routinely used for quality assessment in breeding programs and other industry applications. However, the link between SSC and perceived sweetness may be complex. Discrepancies in the degree of sweetness perception as explained by SSC have been observed across studies (Jouquand et al., 2008; Whitaker et al., 2011; Perez et al., 2016). In addition to genetics, sugar concentration is influenced by harvest dates, locations, maturity, and even fruit-to-fruit variability (Shaw, 1990; Gunness et al., 2009; Hasing et al., 2013). Thus, using ambiguous or arbitrary SSC thresholds may lead to inaccurate conclusions about fruit quality. An ideal predictive framework would take into account uncertainty due to the dynamics of the biological system to construct confidence-based SSC scales. Probabilistic models should facilitate better understanding of biological pathways and mechanisms behind human perception and cognition (Heath et al., 2008; Ma, 2012). In particular, Bayesian models have the advantages of providing hierarchical structures of uncertainty and posterior probability distributions for sample prediction instead of point estimates of means. Advanced computational algorithms that make the implementation of Bayesian models feasible, like Markov chain Monte Carlo simulations, are now available (Van Boekel, 2004; Wilkinson, 2007).

The main objectives of the present study were to (1) combine descriptive sensory analysis and chemical analysis to explore the volatile drivers of sweetness and sourness as well as astringency, green, strawberry, overripe, and woody flavors in fresh strawberries, (2) construct Bayesian models to better define the relationship between sweetness and SSC, and (3) utilize a complex set of strawberry genotypes and environments to better understand the genetic and environmental effects underlying sensory attributes. Each of the three objectives aims to facilitate future flavor breeding by better quantifying factors impacting sensory qualities. Quantifying the effects of individual volatiles will allow us to narrow down the volatile candidates for genetic improvement. Probabilistic SSC evaluation criteria and a better understanding of genotype and environment effects on sensory attributes will inform breeding strategies for sweetness and other sensory qualities.

## Materials and Methods

### Fruit Sampling

In 2009–2010 and 2015–2019, strawberry (*Fragaria × ananassa*) samples from 56 cultivars and advance selections (advance selections were elite breeding lines selected by the breeder, which have been evaluated for other agronomic and quality traits for more than 2 years) (Supplementary Table 1) were harvested two to four times a year, totaling 213 genotypes/harvest date combinations. All samples were harvested from strawberry breeding research plots established at the University of Florida (UF) Gulf Coast Research and Education Center (Balm, FL) or the Florida Strawberry Growers Association headquarters in Dover, FL. All fruiting field trials were arranged in randomized complete block designs and were managed based on recommended commercial practices for Florida strawberry annual plasticulture (Whitaker et al., 2019). At each harvest date, one to five clamshells, depending on fruit availability, of fully ripe fruit from five replicate plots for each genotype were collected and transported to the US Department of Agriculture laboratory in Winter Haven (2009–2010) or Fort Pierce (2015–2019), FL. The fruits were stored at 5°C upon arrival and evaluated 1 day (2009–2010) or 3 days (2015–2019) after harvest. Fruits from each replicate plot were kept separate for chemical analyses but were combined for sensory evaluations.

### Fruit Quality Analysis

Sensory descriptive analysis, SSC, and titratable acidity (TA) methods were previously described (Plotto et al., 2013). In brief, 10 to 12 panelists (three to four males and seven to eight females, age ranging from 25 to 65 years old, with mean range of 41–50 years old) trained to evaluate fresh fruits and fruit products reconvened each year to review descriptors and reference standards used for strawberry evaluation. The fruits were rated on a structured line scale with intervals from 0 to 10, with definitions as follows: 1 to 2 = low, 5 = medium, and 8 to 9 = high intensity of the rated attribute. Reference standards were served at each panel and were for sweet (sucrose 1–5% + citric acid 0.025–0.05%), sour (sucrose 1% + citric acid 0.05–0.15%), astringent (alum 0.125%), strawberry flavor (frozen strawberry puree), green flavor [(*Z*)-3-hexenal in water, 0.5–3.0 ppm], musty/woody (methyl isoborneol, 50 ppb, a drop on filter paper), and fermented/overripe (overripe strawberry left at 25°C overnight) (Plotto et al., 2013). Firmness was not evaluated in 2009 but was added in 2010 and thereafter; no reference was provided for firmness, but the scale was anchored with the words “soft” for ratings 1 and 2 and “firm” for ratings 8 and 9. The fruits were prepared by washing the individual strawberries under running water, drying, and serving as whole fruit (2009–2010) or cut into quarters (2015–2019). Two to three whole fruits or four to eight strawberry quarters were served in individual 4-oz cups with lids (Solo^®^ cup Company, Urbana, IL, United States), making sure that each piece was from a different fruit. The fruits were served at room temperature, in isolated booths under red lighting. Four to six fruit samples (genotypes) were served in one session, at two sessions per day, with up to 12 genotypes randomly distributed across both sessions. Compusense^®^ Five and Compusense Cloud (Compusense Inc., Guelph, ON, Canada) were used to assign a sample presentation following a William’s design pattern and record panelist ratings.

The fruits from each replicate for chemical analyses were different from those used in the sensory panels. Up to 10 fruits (depending on availability) per field replication were cut, with tissue taken for volatile analysis (see below), and the remaining fruits were pureed and frozen at −20°C for later SSC and TA analysis, as described in Plotto et al. (2013).

### Volatile Identification and Quantification

In 2009–2010, 30 g from about 10 fruits per genotype and replication was homogenized for 20 s. Saturated CaCl_2_ was added (w/w) to reduce enzymatic activity (Buttery et al., 1987) right after or at the same time as when homogenizing. Internal standard 3-hexanone (Sigma-Aldrich) was added to a final concentration of 1 ppm. Finally, 5 ml of the mixture was transferred to 20-ml glass vials, crimped with magnetic caps, and stored at −20°C. From 2015 to 2019, 6-g wedges from multiple fruits per genotype were frozen in liquid nitrogen and immediately processed or stored at −80°C. Frozen tissue was grounded with pre-cooled mortar and pestle. Three grams of frozen fruit powder was transferred to 20-ml glass vials (Gerstel) with 3 ml saturated NaCl and 6 μl of internal standard, 3-hexanone at 1,000 ppm, to a final concentration of 1 ppm. The vials were crimped with magnetic caps and stored at −20°C. CaCl_2_ was replaced by NaCl in 2015 after realizing that the calcium from CaCl_2_ might interfere with pectin from the strawberry fruit.

Volatiles were sampled from headspace with a 2-cm tri-phase solid-phase micro-extraction (SPME) fiber (50/30 μm DVB/Carboxen/PDMS; Supelco, Bellefonte, PA, United States) and injected into a gas chromatography–mass spectrometry (GC/MS) system (a model 6890 GC coupled with a model 5973 N MS, Agilent Technologies, Palo Alto, CA, United States) as described by Bai et al. (2014). Briefly, a homogenized sample in the vial was incubated for 30 min at 40°C; the SPME fiber was then exposed to the headspace for 30 min at 40°C. After exposure, the SPME fiber was inserted into the injector of GC to desorb for 15 min at 250°C. A DB-5 (60-m length, 0.25-mm i.d., 1.00-μm film thickness; J&W Scientific, Folsom, CA, United States) column was used, with the oven programmed to increase at 4°C min^–1^, from the initial 40°C to 230°C, and then ramped up at 100°C min^–1^ to 260°C and held for 11.70 min for a total run time of 60 min. Helium was used as the carrier gas at a flow rate of 1.5 ml min^–1^. The settings for MS were inlet, ionizing source, and transfer line temperatures at 250, 230, and 280 °C, respectively. The mass units were monitored from 40 to 250 m/z and ionized at 70 eV.

Volatile identification and quantification of peak areas were conducted with MassHunter Workstation software (Version 10.0; Agilent Technologies). Initial identification was done by mass spectra searches with the NIST library (Version 14, match score > 0.9). The identification was then confirmed by comparing the retention indices generated by running standard C6-C17 alkane mixture under the same conditions as the samples with online resources (NIST Chemistry WebBook and Flavornet.org).

### Bayesian Models of Sweetness Predicted by SSC

Here we described the structure of a robust linear mixed model (Rosa et al., 2003; Svensén and Bishop, 2005):

$$p\left(\beta_{0},\beta_{\text{ssc}},\left\{\tau_{i}\right\},\nu ,\sigma^{2}|\left\{y_{i}\right\},\left\{x_{i}\right\}\right)\propto [\prod_{i}N\left(y_{i}|\beta_{0}+\beta_{\text{ssc}}x_{i},\frac{\sigma^{2}}{\tau_{i}}\right)$$

$$\text{Gamma}\left(\tau_{i}|\frac{\nu }{2},\frac{\nu }{2}\right)]\times N\left(\beta_{0}|0,10\right)N\left(\beta_{\text{ssc}}|0,10\right)Unif\left(\nu |0,100\right)\text{Gamma}\left(\frac{1}{\sigma^{2}}|1,1\right)$$

where β_0_ is the interception, β_ssc_ is the slope of SSC, *y_i_* is the sweetness rating for each sample, *x_i* is the sample SSC, and σ^2^ is the variance of *y_i*. Since the linear model is vulnerable to extreme outliers, robust inference was modeled *via* hyper-parameter τ_*i*_ for changing residual variance in order to reduce the outliers’ influence. All priors for parameters (β_0_,β_ssc_,{τ_*i*_},ν,σ^2^)in the second line were set to random priors.

The above model was extended to a mixed hierarchical model incorporating varying slopes across harvest months.

$$p\left(\beta_{0},\left\{\beta_{j}\right\},\left\{\tau_{i\,j}\right\},\nu ,\sigma^{2},\mu ,\sigma_{1}^{2}|\left\{y_{i\,j}\right\},\left\{x_{i\,j}\right\}\right)\propto [\prod_{j}$$

$$\prod_{i}N\left(y_{i\,j}|\beta_{0}\beta_{j}x_{i\,j},\frac{\sigma^{2}}{\tau_{i\,j}}\right)\text{Gamma}\left(\tau_{i\,j}|\frac{\nu }{2},\frac{\nu }{2}\right)]$$

$$\times \left[\prod_{j}N\left(\beta_{j}|\mu ,\sigma_{1}^{2}\right)\right]N\left(\beta_{0}|0,10\right)N\left(\mu |0,10\right)Unif\left(\nu |0,100\right)$$

$$\text{Gamma}\,\left(\frac{1}{\sigma^{2}}|1,1\right)\,\text{Gamma}\,\left(\frac{1}{\sigma_{1}^{2}}|1,1\right)$$

where β_*j*_ is the slope of SSC at harvest month *j*, and *y*_*ij*_ and *x*_*ij*_ are sweetness score and SSC level of the *i*_*th*_ sample at harvest month *j*, respectively. $\prod_{j}N\left(\beta_{j}|\mu ,\sigma_{1}^{2}\right)$ allows different priors for β_*j*_. The detailed model setup in Just Another Gibbs Sampler 4.3.0 and R software (R version 3.6.3) script to simulate data, run models, and visualize results can be found in the Supplementary Presentation 1.

### Statistical Evaluation

Mean sample sensory attributes were averaged across 10 to 12 panelists (Supplementary Table 3). Chemical data were pooled across field replicates for each sample (Supplementary Table 2). Radar plots were drawn with the “fmsb” package in R software to visualize sensory changes among genotypes and harvest months in 2018 and 2019. Principal component analysis (PCA) and K-clustering (*k* = 3) were conducted on sensory attributes using the “prcomp” and the “kmeans” functions in R for the purpose of visualizing sample and sensory attribute relationships and PCA biplots constructed with the “factoextra” package. In order to find important chemicals that influence sensory attributes, partial least square (PLS) models were built for each year with all chemical data and sensory firmness from the DA to account for mouth feel since fruit firmness was not instrumentally measured, and pH was excluded due to its high correlation with TA. PLS (number of components = 5) was analyzed with the “plsr” function and “PLSVarSel” package in R. A chemical was deemed important if the variable importance for the projection (VIP) (Chong and Jun, 2005) was larger than 1. The importance index used to compare importance among chemicals was built such that importance index = (number of years with VIP > 1 and positive effect) – (number of years with VIP > 1 and negative effect). The range of index was anchored at ±7, allowing chemicals to have significant positive or negative effects for all 7 years of data. To investigate genetic and environmental effects on sensory and physicochemical attributes, individual multivariate models with fixed effects, *YGMGMe*, were built for each year and attribute, where *Y* is a sensory attribute, *G* is the genotype effect, *M* is the harvest month effect, and *G* × *M* represents their interaction. The *P*-values for all effects were then extracted with the ANOVA function in R. Negative log transformed *p*-values, after Bonferroni correction, were plotted to show the significance level for each effect.

## Results and Discussion

### Descriptive Analysis of Fresh Strawberry Fruits

**FIGURE 1:**
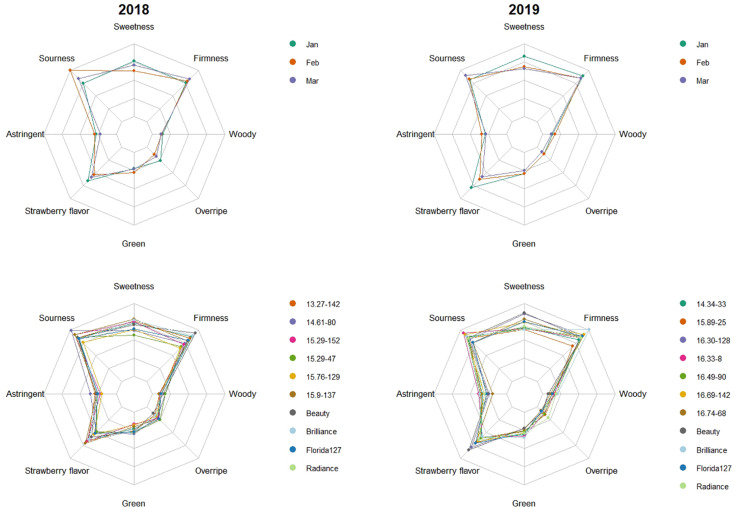
Genotype means across harvest months and harvest month means across genotypes in 2018 and 2019 for eight descriptive sensory attributes. The top two plots show harvest month means across genotypes. The differently colored lines represent months. The bottom two plots show genotype means across three harvests. Colored lines represent genotypes. Scale from 0 to 10 was used for all attributes.

**FIGURE 2:**
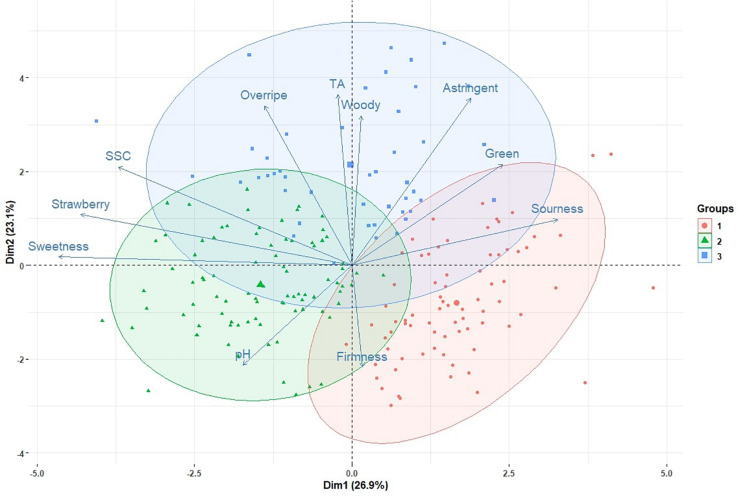
Biplot with the first two components from a principal component analysis. Vectors represent sensory and physiochemical attributes. Points represent individual samples. Three colored eclipses indicate three clusters from a K-cluster analysis.

In the 2009, 2010, 2015, 2016, 2017, 2018, and 2019 seasons, a total of 213 genotype/harvest date combinations from 56 cultivars and advanced selections were subjected to DA. The mean sweetness of all samples was 4.2, with a range from 2.5 to 5.9, on a 0-to-10 scale (Supplementary Table 3). Among all descriptors, the smallest range was observed for woody flavor, from 0.2 to 2.2 (Supplementary Table 3). Substantial eating quality differences were observed among genotypes. In 2018 and 2019, “Florida Beauty” had the highest sweetness (average 5.0), while ‘Florida Brilliance’ had the highest sensory firmness (average 5.8) (Figure 1). Decreasing average sweetness was observed from January to March in each year, in line with previous findings (Jouquand et al., 2008; Fan et al., 2020). This decline in sweetness was reflected by a similar decline in SSC due to rising temperatures during the Florida fruiting season (MacKenzie and Chandler, 2009; Hasing et al., 2013). Since changes in temperature alter enzymatic activity during fruit development, volatiles also exhibited great variability over the harvest months. In extreme cases, presence-or-absence changes were observed for some volatiles (Supplementary Table 2). Thus, a similar decline was also observed for strawberry flavor (Figure 1). The large variability of volatiles emphasizes the importance to evaluate the sensory qualities of strawberry over multiple harvests and seasons. PCA with two components using sensory descriptors, SSC, TA, and pH explained 50% of the total variation (Figure 2). Sweetness and strawberry flavor, which are desirable sensory attributes, as well as SSC, were negatively correlated with PC1, in contrast with sourness and green flavor. PC2 was positively correlated with TA, overripe, and woody flavors, which were undesirable traits and were negatively correlated with firmness. Similar PCA patterns have been observed for multiple DA studies using fresh strawberries (Plotto et al., 2013; Oliver et al., 2018a). The samples could be grouped into three clusters using K-means clustering (Figure 2 and Supplementary Table 1). The first cluster was mostly confined to the 4th quadrant with high sourness and green flavor. The second cluster was along the negative side of PC1, classified with high sweetness, strawberry flavor, and firmness. This cluster appeared to comprise samples with the most desirable attributes. Our recent releases, “Florida Beauty” and Sweet Sensation^®^ “Florida127,” were consistently clustered in group 2. Specifically, “Florida Beauty” was rated group 2 in 10 out of 14 evaluations and Sweet Sensation^®^ ‘Florida127’ was rated group 2 in 13 out of 16 evaluations (Supplementary Table 1). The last cluster was correlated with higher levels of woody flavor, overripe flavor, and astringent mouthfeel. While there is fluctuation over the years, sweetness and strawberry flavor have always been strongly correlated. In contrast, sourness has been correlated with astringent mouthfeel, green, woody, or overripe flavors, depending on the season (Supplementary Presentation 2 and Plotto et al., 2013).

During the breeding process, many criteria are weighted to select the most well-rounded genotypes. Improvement of eating quality is generally subject to the taste preferences and limited sampling by the breeding team. DA provides a quantitative tool to finely discriminate the eating quality of different genotypes. Since consumer acceptance can be inferred from desirable attributes like sweetness, strawberry flavor, and firmness (Azodanlou et al., 2003), a DA-based quality index has been developed and implemented for strawberry growers and breeding programs (Ares et al., 2009). However, caution must be taken when extrapolating the index across programs because of differences in understanding of the scale and consumer expectations. To maximize the impact of DA, the process of identifying descriptors, developing quality indices, and establishing benchmarks must be carefully undertaken.

### Volatiles Modulating Sensory Attributes

The chemical analysis of 213 samples yielded 71 volatiles, covering 17 of 22 aroma-active volatiles reported in Nuzzi et al. (2008) and 21 of 29 aroma-active volatiles evaluated with GC–olfactometry in ’Du et al. (2011). Volatiles with significant influence on sensory attributes were identified with PLS models. In order to minimize the effect of sample preparation and environments, the PLS model was analyzed independently for each year, and the summary statistic of importance index was used to gauge the overall effect on sensory attributes across 7 years. Models using only 5 years of data from 2015 to 2019 identified a consistent but smaller set of important chemicals. A significant effect of a volatile for a sensory quality had to be consistent over at least 2 years to be considered as influential.

**FIGURE 3:**
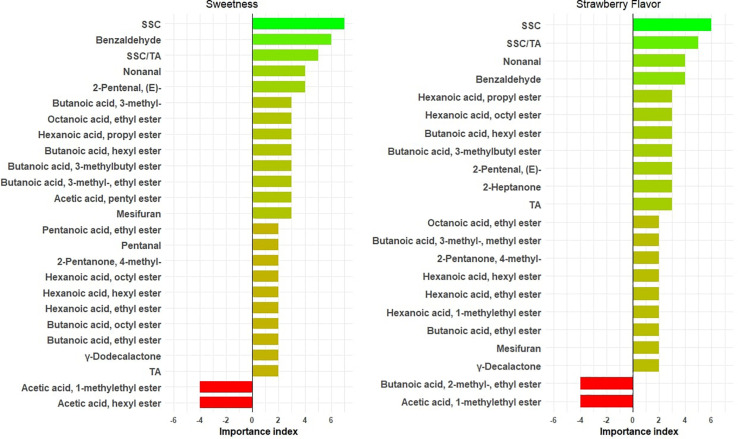
Important physicochemical measures/volatiles for sweetness and strawberry flavor. The *X* axis shows importance index = [number of years with variable importance in projection (VIP) > 1 and positive effect] – (number of years with VIP > 1 and negative effect). The bars are annotated with chemical names.

Twenty volatiles were shown to enhance sweetness independent of SSC (Figure 3 and Supplementary Table 4). While only 14 genotypes overlapped with our previous consumer study (Supplementary Table 5 and Fan et al., 2020) and the volatile sampling methods were different, our top three sweetness-enhancing volatiles [benzaldehyde, 2-pentanal, (E) and nonanal] were identified with both consumer and DA panels. 2,5-Dimethyl-4-methoxy-3(2H)-furanone (mesifurane), butanoic acid/ethyl ester, and hexanoic acid/ethyl ester exhibited a sweetness-enhancing ability in multiple years (Figure 3), in line with findings based on odor threshold and odor active values (Pyysalo et al., 1979; Larsen and Poll, 1990; Ulrich et al., 1997). In agreement with the previous consumer study, two medium-chain butanoic acid esters (butanoic acid, hexyl ester and butanoic acid, and octyl ester) had increased sweetness, as well as three medium-chain hexanoic acid esters (hexanoic acid, hexyl ester; hexanoic acid, octyl ester; and hexanoic acid, propyl ester). Butanoic acid, 3- methyl-, and two derived esters (butanoic acid, 3- methyl-, ethyl ester, and butanoic acid, 3-methylbutyl ester) had positive effects on sweetness and have been identified in multiple GC-O studies (Ulrich et al., 1997; ’Du et al., 2011; Cannon et al., 2015). Surprisingly, hexyl acetate, one of the most potent esters (’Du et al., 2011) in strawberry, had a negative effect on sweetness, possibly related to its green apple aroma at higher concentrations (Young et al., 1996). Volatiles influencing strawberry flavor mostly overlapped with sweetness influencers (Figure 3). Furanones, esters, and lactones generally not detected in white or half-red fruit undergo dramatic increases in the late stages of ripening (Ménager et al., 2004), shaping the strawberry flavor in ripe fruit. A major determinant in peach aroma, γ-decalactone, contributed to strawberry flavor, but not sweetness, in this study. The only volatile found to decrease both sweetness and strawberry flavor was acetic acid, 1-methylethyl ester.

Soluble solids content unsurprisingly had the highest correlations with both sweetness and strawberry flavor. Sugar content is also the main determinant for consumer liking (Schwieterman et al., 2014). Given the potential yield cost imposed by breeding varieties with higher sugar level (Whitaker et al., 2012), manipulating volatiles may provide a better alternative for the enhancement of eating quality. In our previous consumer sensory study, we found that most sweetness enhancers overlapped strongly with liking enhancers (Fan et al., 2020). In the present study, the prior list of sweetness-enhancing volatiles based on consumer studies is expanded with new compounds such as mesifurane; butanoic acid, ethyl ester and hexanoic acid, ethyl ester; and butanoic acid, 3- methyl-, which have been historically considered as important volatiles for strawberry flavor, highlighting the diverse flavor profiles existing in commercial germplasm.

**FIGURE 4:**
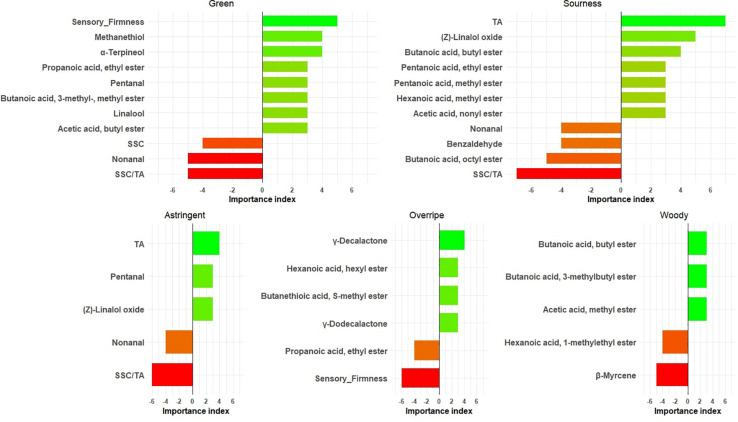
Important physicochemical measures/volatiles for five sensory attributes. The *X* axis shows the importance index [number of years with variable importance in projection (VIP) > 1 and positive effect] – (number of years with VIP > 1 and negative effect). The bars are annotated with chemical names.

In this study, alpha-terpineol; methanethiol; acetic acid, butyl ester; linalool; butanoic acid, 3- methyl-, methyl ester; pentanal; and propanoic acid, ethyl ester were identified as green flavor contributors (Figure 4 and Supplementary Table 4). Aldehydes like pentanal, hexanal, and 2-hexenal, (*E*)- have typically been linked to green notes in immature fruit (Perez et al., 1992; Ménager et al., 2004) but are much reduced and suppressed by other strawberry aromas upon ripening (Jetti et al., 2007). Unsurprisingly, firmness was found to be related to green notes (Figure 4). TA was the major contributor for sourness in all 7 years (Figure 4). Besides TA, (Z)-linalool oxide appeared to have positive effects on sourness, as opposed to benzaldehyde and nonanal. TA was also highly correlated with astringent mouthfeel, but not as strongly as for the sourness–TA relationship (Figure 4). Perceived astringency of fruit is mainly associated with phenolic compounds (Joslyn and Goldstein, 1964) and acids (Lawless et al., 1996). Two lactones (γ-decalactone and γ-dodecalactone) and butanethioic acid, S-methyl ester were associated with overripe flavor (Figure 4). During post-harvest storage, volatile compositions undergo large changes (Lu et al., 2018), such that the higher relative abundances of γ-decalactone may be observed in overripe strawberries. Since sulfur esters are usually described as giving undesirable aromas (Du et al., 2011), it is not surprising that butanethioic acid, S-methyl ester appears to be one of the compounds contributing to overripe flavor, which was also defined as “fermented.” Because our study includes fruit from the narrow genetic pool of the UF strawberry breeding program, it does not present an exhaustive list of volatiles influencing sensory attributes from strawberry. Future identification of new sensory-modulating volatiles should embrace a wider germplasm and the wild relatives of cultivated strawberry.

### Modeling Sweetness Based on Physiochemical Parameters

**FIGURE 5:**
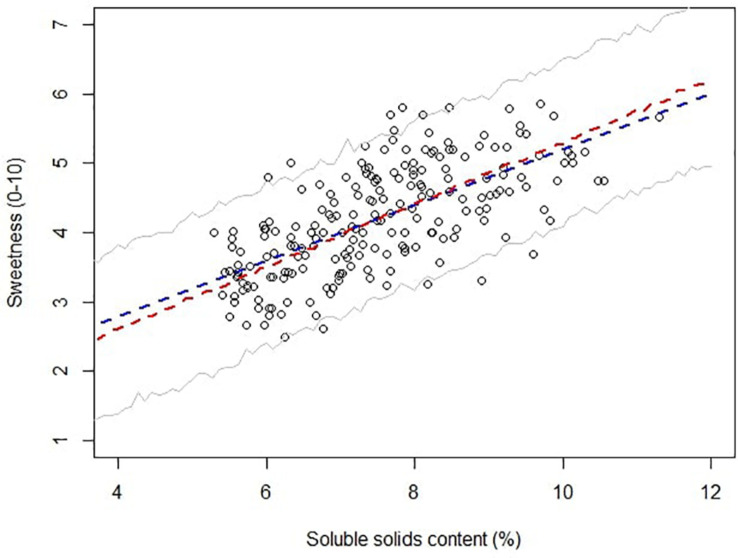
Scatter plot of sweetness against soluble solids content. The blue line represents least square linear regression, and the red line represents the mean Bayesian robust regression. Gray lines represent 95% confidence intervals for posterior distributions.

**FIGURE 6:**
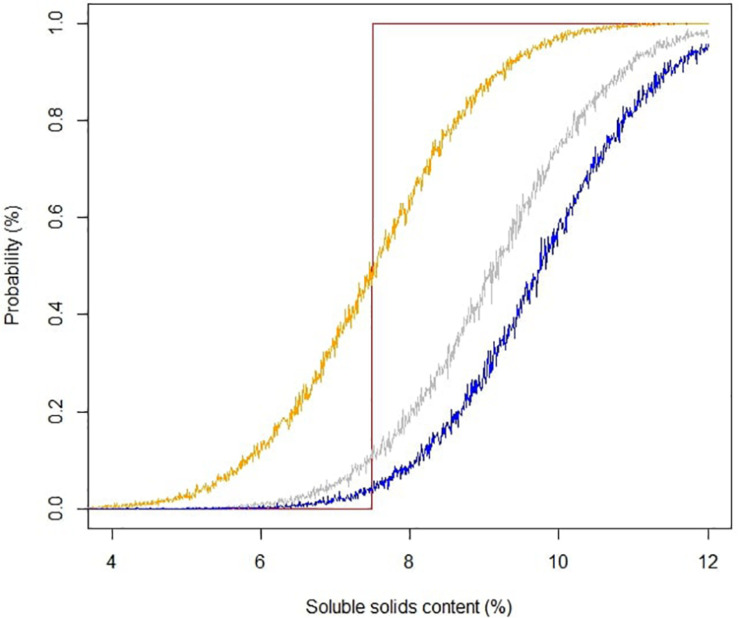
Probability of sweetness perception based on soluble solids content. Yellow, gray, and blue lines represent accumulated probability distributions of above average, top 20%, and top 10% sweetness, respectively. The red line represents the inference from a least square linear model.

Soluble solids content is a robust analytical measurement that strongly correlated with total sugars in strawberry (Jouquand et al., 2008; Gunness et al., 2009). The initial implementation of SSC in strawberry quality control and breeding can be traced back to the late 1980s, and it is still routinely used in the strawberry industry (Alavoine and Crochon, 1988; Ares et al., 2009). In Alavoine’s report, SSC-based thresholds were proposed to distinguish medium taste quality from high taste quality, with an arbitrary cutoff for the highest taste quality placed at SSC of 8%. Thirty years have passed, and commercial strawberry quality has seen an improvement. Some current cultivars like “Florida Beauty” had an average SSC of 8.2% across all harvests in this study. To guide future benchmarks, we utilized a Bayesian robust model to fit SSC against sweetness perception due to its ability to incorporate sample uncertainty and parameter uncertainty. Our model successfully converged with average $\hat{R}$ smaller than 1.1. The mean of the marginal posterior distribution slope β_ssc_ was 0.45, with SD of 0.03 and 95% credibility from 0.40 to 0.50, indicating a strong positive correlation between SSC and sweetness. The 95% credible marginal posterior distribution of y (Figure 5) captured 196 of 207 samples (94.7%). Eleven outliers were weighted less in the model with τ_*i*_ smaller than 1, ranging from 0.91 to 0.96. The main goal of adopting a Bayesian model was to give probabilistic-based predictions of sample sweetness from SSC data for breeding and quality control purposes. Striking differences for the probability of greater-than-average sweetness was obtained after incorporating uncertainty into the model. Predictions without uncertainty indicated that samples with SSC higher than 7.5% would have greater-than-average sweetness intensity (Figure 6). However, the Bayesian model indicated a 50% chance of greater-than-average sweetness at SSC of 7.5%. To achieve 80% confidence, SSC must be higher than 8.6%. A simplified probability chart with SSC ranging from 6 to 12 is shown in Table 1.

**TABLE 1:** Sweetness probability chart for levels of soluble solids content (SSC).

SSC (%)	Sensory sweetness probability		
	
	Above average	Top 20%	Top 10%
6.00	0.11^a^	0.01	0.01
6.50	0.22	0.04	0.01
7.00	0.35	0.04	0.02
7.50	0.50	0.08	0.06
8.00	0.64	0.20	0.08
8.50	0.77	0.30	0.17
9.00	0.86	0.48	0.29
9.50	0.94	0.58	0.42
10.00	0.97	0.74	0.58
10.50	0.99	0.83	0.72
11.00	1.00	0.92	0.83
11.50	1.00	0.96	0.89
12.00	1.00	0.98	0.96

*^*a*^The probability of above-average sweetness perception at a soluble solids content of 6% is 11%.*

**FIGURE 7:**
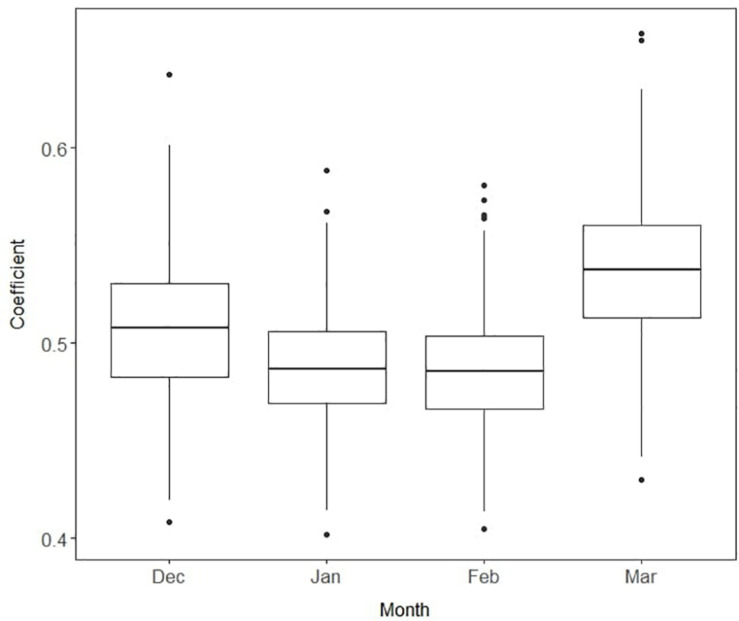
Boxplots of the slope coefficients for soluble solids content by month. Each box represents median and interquartile range from 15,000 iterations. Higher values imply higher correlations of soluble solids content with sensory sweetness.

During winter and spring production in a subtropical climate, strawberry SSC exhibits large within-season variations due to changes in plant physiological and environmental conditions (MacKenzie and Chandler, 2009). Differences in SSC stability over the fruiting season among genotypes add additional complications to the selection criteria for sweetness (Hasing et al., 2013). Furthermore, the relationship between SSC and sweetness can be strongly influenced by environmental conditions (Azodanlou et al., 2003). To examine the stability of relationship between SSC and sweetness during the season, we introduced varying slopes β_*j*_ to the Bayesian model, which allowed heterogeneity of the sweetness–SSC relationship. The highest coefficient of the slope was found in March, with a mean of 0.54 and 95% confidence interval of 0.47 to 0.61 (Figure 7). Lower coefficients were found in January and February, around 0.49. Our results suggest that March was the best month to make selections for SSC due to its higher correlation with perceived sweetness. This is also consistent with the objective of maintaining fruit quality at the conclusion of the season when conditions are more unfavorable for eating quality (Hasing et al., 2013). Breeders may therefore consider reducing the number of SSC phenotyping events early in the season to save labor and resources or more heavily weighting late-season observations.

### Genetic and Environmental Factors Influencing Sensory and Physicochemical Attributes

**FIGURE 8:**
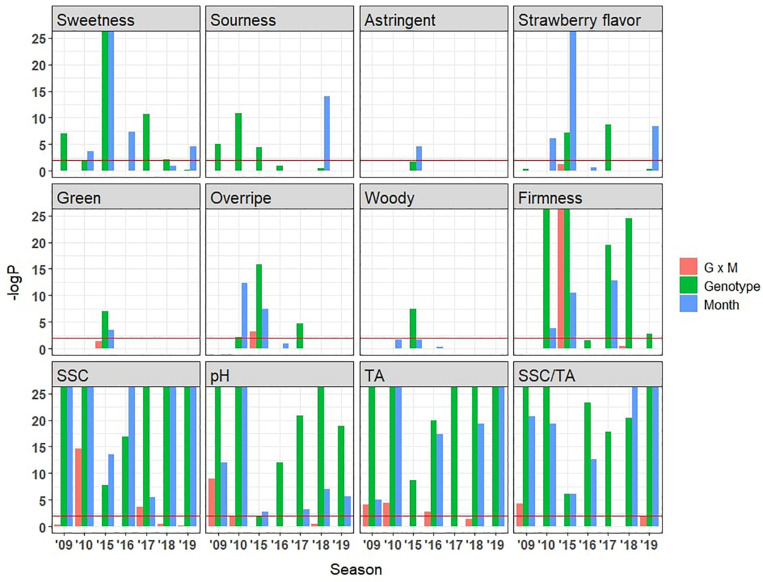
Influence of genotype, harvest month, and month by genotype interaction effects on sensory and physiochemical attributes as indicated by –logP values. Horizonal lines indicate –logP values of 2 (Bonferroni-corrected *p*-value = 0.01). Taller bars were observed for physicochemical attributes compared to sensory attributes due to low variance among field replicates compared to panelist variance.

The chemical compositions of strawberries are strongly affected by both genotypes and growing conditions (Forney et al., 2000), leading to eating quality differences (Jouquand et al., 2008). However, the existing literature exploring the fluctuation of sensory characteristics based on genotypes or environments only encompasses a few genotypes grown in one or two seasons (Jetti et al., 2007; Jouquand et al., 2008). In the present study, effects from genotype, harvest month, and their interactions were compared across 7 years (Figure 8). Sweetness was controlled by both harvest month and genotype, with no significant interaction between the two. Genotype was significant in 5 years, and harvest month was significant in 4 years. This result is corroborated by significant genotype and month effects for SSC in six of 7 years. Fewer genotype and month effects were observed for strawberry flavor. Sourness was mainly controlled by genotype, consistent with greater significance for genotype than for month for TA and pH (Figure 8). Sensory firmness also appears to be mainly controlled by genotype. Overripe flavor showed significance for genotype in 3 years and for harvest month in 2 years. Astringent, green, and woody flavors had little influence from genotype or month. There were no consistent G × E interactions for any sensory attributes. Although no previous sensory studies have included such large numbers of genotypes and environments (Ulrich et al., 2018), metabolite surveys have shown moderate to high heritability for SSC and volatile abundance (Whitaker et al., 2012; Gezan et al., 2017; Urrutia et al., 2017). A large portion of variability in most volatiles found in wild strawberry is explained by one or two major QTLs (Urrutia et al., 2017). Consistent with these studies, genetics was the primary force driving variation in volatile abundances among our strawberry samples (Supplementary Presentation 3). As expected, environmental effect is also pervasive for most volatiles over the harvest months (Supplementary Presentation 3). Our results indicate the strong genetic control of firmness, sourness, sweetness, and strawberry flavor, implying further potential for genetic improvement of these sensory attributes in the germplasm tested.

### A Paradigm for Sensory Quality Improvement in Strawberry

Increasing consumer liking is one of the main goals of strawberry breeding. Descriptive analysis provides an objective evaluation of sensory characteristics that are strongly associated with consumer preference. Sweetness and strawberry flavor contribute to liking, while sour, astringent, overripe, and green flavors detract from liking (Oliver et al., 2018b). At the intermediate stage of cultivar development, elite selections are grown in small, replicated trials for quality evaluation. Fruit yield from those trials is too small to supply large consumer panels (Fan et al., 2020), but small trained panels are well suited for this purpose. Importantly, these data also allow sensory evaluations to drive targeted genetic solutions for improving eating quality, for example, γ-decalactone was identified as a flavor target based on sensory evidence (Schwieterman et al., 2014) and the identification of a candidate gene (*FaFAD1*) responsible for the presence and abundance of this volatile (Chambers et al., 2014; Sánchez-Sevilla et al., 2014; Noh et al., 2017). An improved codominant DNA maker for *FaFAD1* was recently designed, validated, and implemented in a marker-assisted selection (Oh et al., 2019). Thus, a fruit quality improvement paradigm that begins with consumer-desired sensory attributes and progresses to chemical targets and finally to genetic tools is now feasible in strawberry. This paradigm is applicable not only in strawberry but also to a broad array of crops valued for their sensory qualities.

In conclusion, we identified additional volatiles that enhance sweetness independently of sugars, such as benzaldehyde; 2-pentenal, (E)-; nonanal; mesifurane; butanoic acid, 3- methyl-, ethyl ester; butanoic acid, 3-methyl-; γ-dodecalactone; butanoic acid, ethyl ester; and hexanoic acid, ethyl ester, many of which have been historically identified as important to strawberry flavor and in our consumer study (Fan et al., 2020). Thus, the identification of these volatiles will allow us to narrow down to a smaller number of flavor breeding targets. Sweetness prediction based on SSC has been updated using a probabilistic approach, better informing strategies for improving strawberry sweetness. Lastly, our quantification of genotype and environment effects and their interaction on various sensory attributes provides a statistical basis for the strategic improvement of strawberry sensory quality.

## Data Availability Statement

The original contributions presented in the study are included in the article/Supplementary Material, further inquiries can be directed to the corresponding author.

## Ethics Statement

Ethical review and approval was not required for the study on human participants in accordance with the Local Legislation and Institutional Requirements. Written informed consent for participation was not required for this study in accordance with the National Legislation and the Institutional Requirements.

## Author Contributions

ZF performed data analyses and composed the manuscript. VW and AP designed the study and oversaw the whole project. JB organized and performed chemical analyses. AP organized and performed descriptive sensory panels. All the authors read and approved the final manuscript.

## Conflict of Interest

The authors declare that the research was conducted in the absence of any commercial or financial relationships that could be construed as a potential conflict of interest.

## Supplementary Material

Supplementary Presentation 1 R-script to simulate data, generate JAGS models and visualize results.

Supplementary Presentation 2 PCA plots of sensory attributes for individual year.

Supplementary Presentation 3 Influence of genotype, harvest month, and month by genotype interaction effects on volatile abundance as indicated by −logP values.

Supplementary Table 1 Sample basic information and cluster based on sensory attributes. Four columns list sample genotype, harvesting year, month, and cluster number. NA means the sample was either not sent for sensory evaluation, or not quantified for TA and SSC.

Supplementary Table 2 Chemical data for each sample. CAS numbers were provided.

Supplementary Table 3 Average sensory attributes across panelists and average SSC, pH, and TA across sample replicates. NA means not measured.

Supplementary Table 4 Importance index of all volatiles. Other tabs include PLS results for individual sensory attributes, including VIP scores and coefficients for each volatile.

Supplementary Table 5 Summary of common genotypes used in this study and the previous consumer study (Fan et al., 2020).
