# Supplementary material for: Volatiles Influencing Sensory Attributes and Bayesian Modeling of the Soluble Solids–Sweetness Relationship in Strawberry
Source: Front Plant Sci. 2021 Mar 17;12:640704. doi: 10.3389/fpls.2021.640704 (PMC8010315; doi:10.3389/fpls.2021.640704)
Supplement: Supplementary Presentation 1 — R-script to simulate data, generate JAGS models and visualize results. [file Presentation_1.PDF]

Supplementary Material

Zhen Fan

5/4/2020

JAGS models

Robust Bayesian model

```
model{
  for (i in 1:N){
    tau[i] ~ dgamma(v/2,v/2)
    y[i] ~ dnorm(b0 + b1 * x[i], prec*tau[i])
  }
  b0 ~ dnorm(0,10)
  b1 ~ dnorm(-1,10)
  prec ~ dgamma(1,1)
  v ~ dunif(0,100)
}
```

Hierachical model with varing slopes

```
model{
  for (j in 1:m) {
    b1[j] ~ dnorm(mu,prec1)
  }
  for (i in 1:N){
    tau[i] ~ dgamma(v/2,v/2)
    y[i] ~ dnorm(b0 + b1[Month[i]] * x[i], prec*tau[i])
  }

  b0 ~ dnorm(0,10)
  mu ~ dnorm(0,10)
  prec ~ dgamma(1,1)
  prec1 ~ dgamma(1,1)
  v ~ dunif(0,100)
}
```

Create simulation and test it with our model

To test the credibility of our models, we generated simulated data. Our simulation created varing slopes, outliers and corresponding Ys.

```
library(jagsUI)
library(tidyverse)
set.seed(156)

# MCMC settings
ni <- 15000 #number of iterations
nt <- 50    #interval to thin
nb <- 5000  #number of iterations to discard as burn-in
nc <- 3     #number of chains
```

```
b1 = c(0.3,0.4,0.5,0.6) #Four different slopes
sd = 1
b0 = 0
x = runif(200,min = 5, max = 13) #random x from range (5,13)
month = rep(c(1,2,3,4),50)
y = rnorm(200, mean = b0+b1*x, sd = sd)
m = max(month)
params=c("b0","b1",'prec','v','tau','mu','prec1')
y[1:3] = c(10,11,13)
```

```
model3=jags(model.file="sweetness_randomslope_Model.R",
            parameters.to.save=params,data=list(y=y,N=length(x),x=x,m=m,Month=month),n.chains=nc,
            n.burnin=nb,n.iter=ni,n.thin=nt,DIC=TRUE)
```

Results were plotted across iterations. Horizonal lines are true parameters.

```
model3$summary[1:11,]
```

```
##                mean         sd         2.5%         25%         50%        75%
## b0      -0.01032485 0.21080925 -0.428381875 -0.16079281 -0.01701194 0.14272262
## b1[1]    0.31080656 0.02658081  0.260428347  0.29293299  0.31129622 0.32968995
## b1[2]    0.39655904 0.02811204  0.341011015  0.37746738  0.39710723 0.41552560
## b1[3]    0.50826077 0.02826125  0.456145783  0.48853096  0.50759077 0.52820232
## b1[4]    0.59033342 0.02833644  0.536830103  0.57155600  0.59046621 0.60778881
## prec     1.26653234 0.19827100  0.927449783  1.13765941  1.25035139 1.38452709
## v        3.93436894 1.00172132  2.470949325  3.24988076  3.78494240 4.44966068
## tau[1]   0.05690199 0.04411474  0.006711400  0.02562456  0.04754357 0.07593907
## tau[2]   0.05632765 0.04032759  0.006912774  0.02713852  0.04532572 0.07675083
## tau[3]   0.03904957 0.02846582  0.004858249  0.01852859  0.03266997 0.05222193
## tau[4]   1.21905918 0.74964672  0.195899907  0.65802482  1.09645205 1.60777405
##             97.5%      Rhat n.eff overlap0    f
## b0      0.4033546 1.0045296   552        1 0.53
## b1[1]   0.3604489 1.0048316   331        0 1.00
## b1[2]   0.4482875 1.0092625   367        0 1.00
## b1[3]   0.5591490 0.9993680   600        0 1.00
```

```
## b1[4]   0.6465383 0.9996056   600        0 1.00
## prec    1.6923634 1.0009252   600        0 1.00
## v       6.3040193 1.0096278   600        0 1.00
## tau[1]  0.1598862 1.0235786   519        0 1.00
## tau[2]  0.1537152 1.0025396   600        0 1.00
## tau[3]  0.1048157 1.0028569   600        0 1.00
## tau[4]  3.1792124 0.9989384   600        0 1.00
```

```r
par(mar = c(5,5,5,2),mfcol=c(2,2),cex.lab=2)
plot(model3$sims.list$b1[,1],ylab = 'b1_1',type = 'l');abline(h=0.3,col = 'blue')#trace plot for b1
plot(model3$sims.list$b1[,2],ylab = 'b1_2',type = 'l');abline(h=0.4,col = 'blue')
plot(model3$sims.list$b1[,3],ylab = 'b1_3',type = 'l');abline(h=0.5,col = 'blue')
plot(model3$sims.list$b1[,4],ylab = 'b1_4',type = 'l');abline(h=0.6,col = 'blue')
```

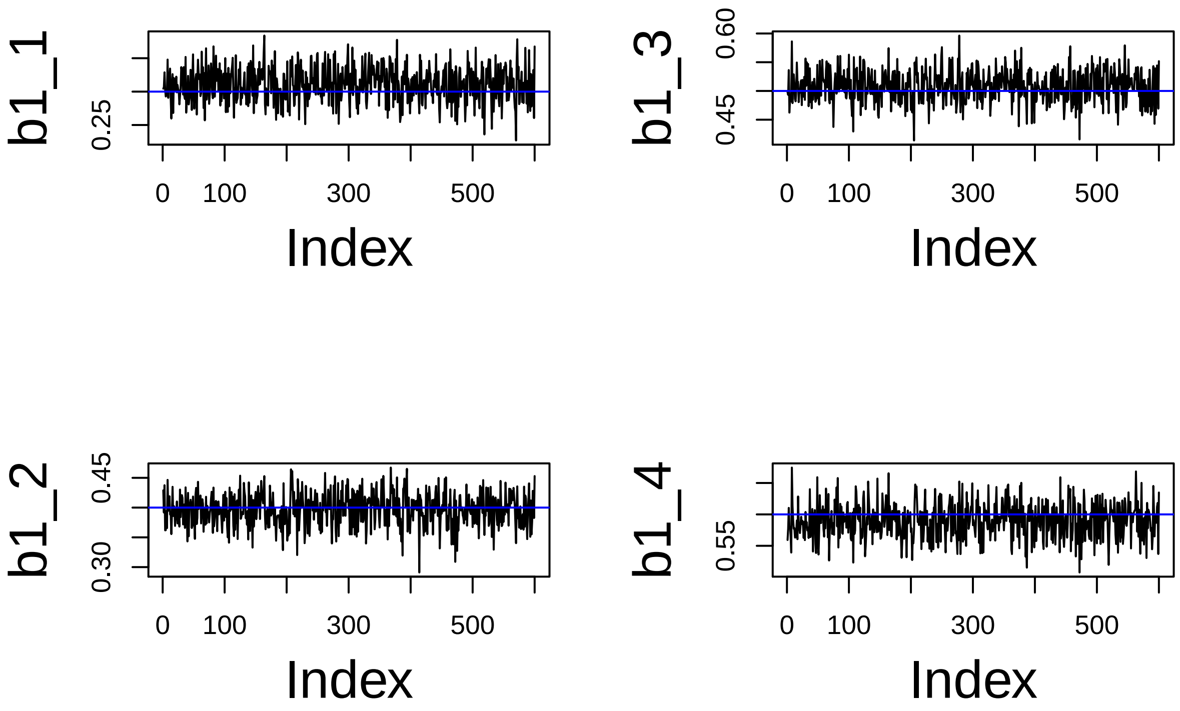

Our model successfully retrieved assigned four slopes and gave low weights to the outliers.

```r
par(mar = c(5,5,5,2),mfcol=c(1,1),cex.lab=2)
mean_tau = apply(model3$sims.list$tau, 2 , mean); plot(mean_tau,type = 'hist');abline(h=1,col = 'blue')
```

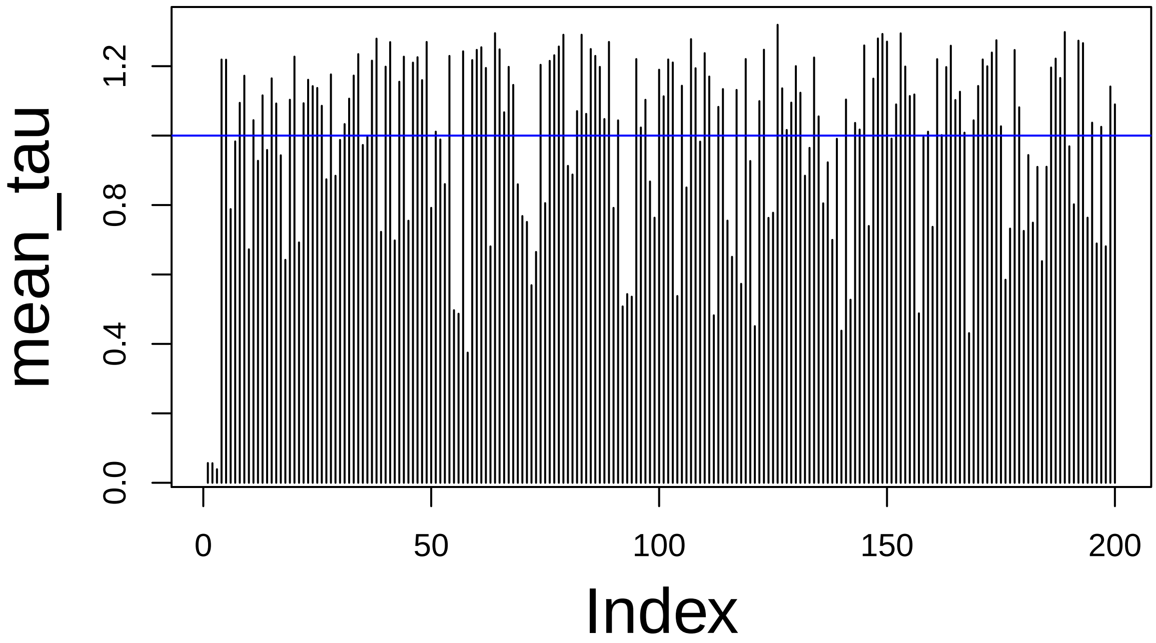

mean_tau
0.0
0.4
0.8
1.2
0
50
100
150
200
Index
