# Supplementary figures and images for: Volatiles Influencing Sensory Attributes and Bayesian Modeling of the Soluble Solids–Sweetness Relationship in Strawberry
Source: Front Plant Sci. 2021 Mar 17;12:640704. doi: 10.3389/fpls.2021.640704 (PMC8010315; doi:10.3389/fpls.2021.640704)

# Presentation 2. PCA plots of sensory attributes for individual year.

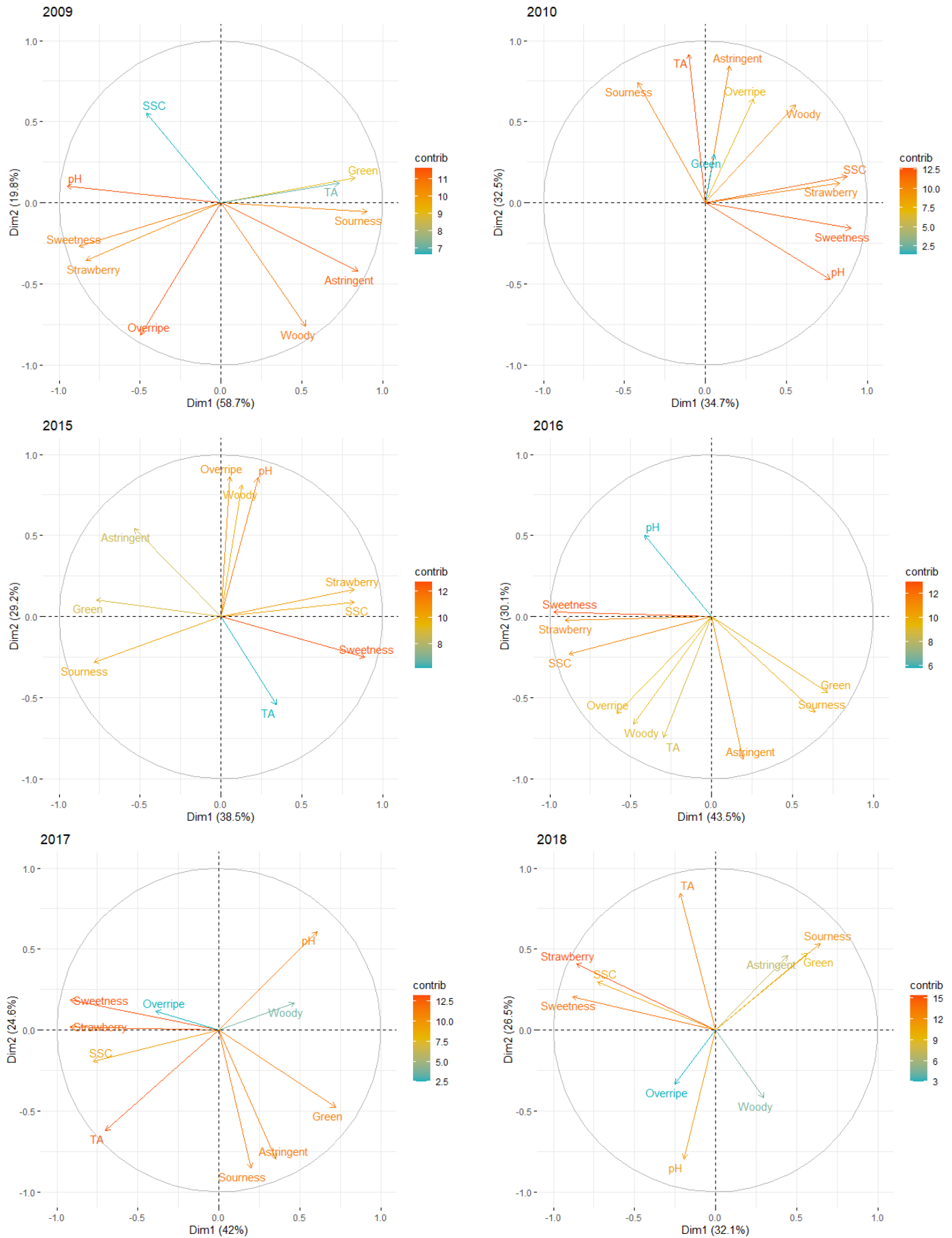

2019

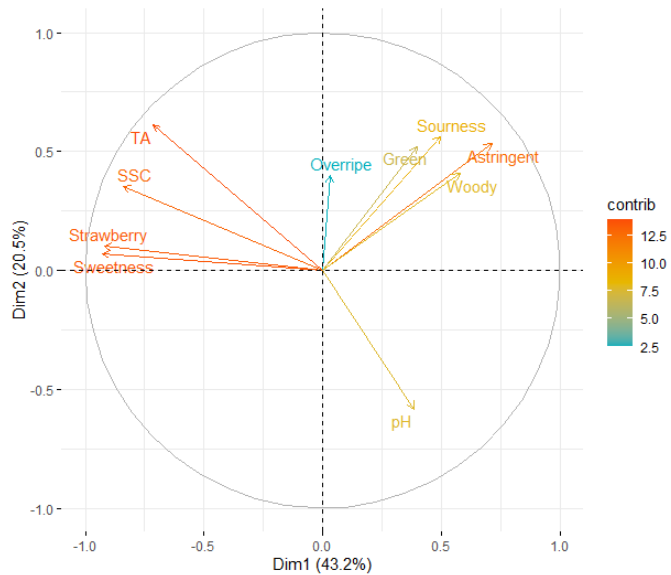

Supplement: Supplementary Presentation 2 — PCA plots of sensory attributes for individual year. [file Presentation_2.PDF]

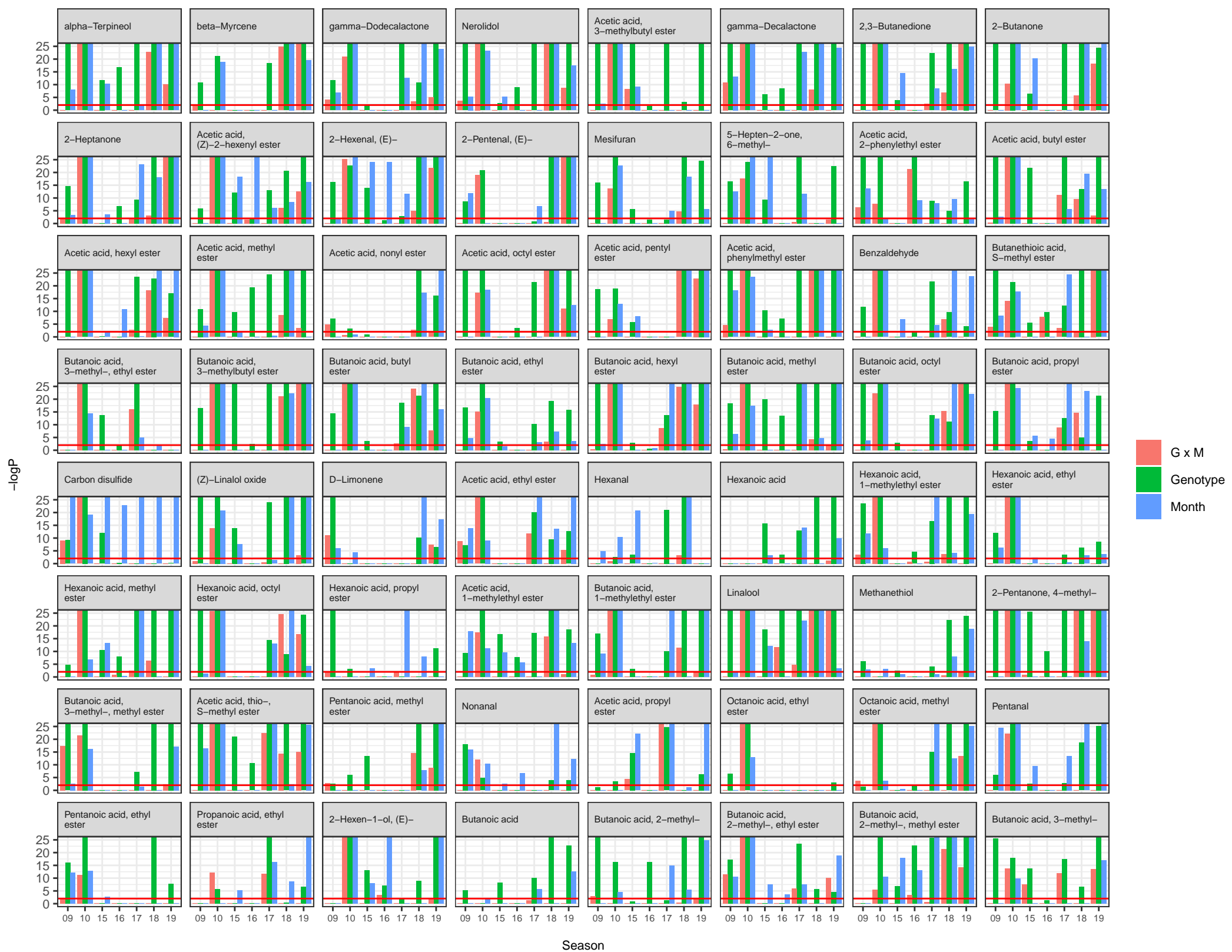

Supplement: Supplementary Presentation 3 — Influence of genotype, harvest month, and month by genotype interaction effects on volatile abundance as indicated by −logP values. [file Presentation_3.PDF]
